# Structural Polymorphism of polyG Inclusions Revealed by In Situ Cryo‐Electron Tomography

**DOI:** 10.1002/advs.76603

**Published:** 2026-07-14

**Authors:** Yunwen Qian, Yalan Wan, Chen Chen, Xiaoyu Zheng, Wenjing Du, Junhan Yang, Zhihao Quan, Biyu Yang, Jiaxi Yu, Jie Zheng, Zhaoxia Wang, Jianwen Deng, Qiang Guo

**Affiliations:** ^1^ State Key Laboratory of Membrane Biology Peking‐Tsinghua Joint Center For Life Sciences Academy for Advanced Interdisciplinary Studies School of Life Sciences Peking University Beijing China; ^2^ Department of Neurology Peking University First Hospital Beijing China; ^3^ Rare Diseases Medical Center Peking University First Hospital Beijing China; ^4^ Changping Laboratory Beijing China; ^5^ Neuroscience Research Institute and Department of Neurobiology School of Basic Medical Sciences Peking University Beijing China; ^6^ Key Laboratory for Neuroscience Ministry of Education/National Health Commission Peking University Beijing China

**Keywords:** cryo‐ET, neurodegeneration, neuronal intranuclear inclusion disease, polyG diseases

## Abstract

NIID (Neuronal intranuclear inclusion disease) is defined by ubiquitin‐ and p62‐positive intranuclear inclusions, yet their native ultrastructure remains unclear. Using correlative cryo‐electron tomography in primary cortical neurons and brain tissue from an NIID mouse model, we show that polyG inclusions are built from interconnected ribbon‐like assemblies rather than canonical amyloid fibrils. PolyG populates multiple compartment‐specific ribbon states, including a nuclear ribbon network enriched in 26S proteasomes and two cytoplasmic ribbon packing states with sharply different proteasome accessibility. In the cytoplasm, ribbon assemblies frequently contact endomembranes—particularly ER‐like membranes—and these interactions coincide with membrane deformation, consistent with transcriptomic dysregulation of ER‐stress responses‐related genes. Together, these findings establish multiple ribbon states as a core feature of polyG aggregation and provide an in situ framework for linking NIID inclusion architecture to cellular interactions.

## Graphical abstract

Correlative cryo‐electron tomography in primary cortical neurons and NIID mouse brain tissue reveals that polyG inclusions are interconnected ribbon‐like assemblies rather than canonical amyloid fibrils. Multiple compartment‐specific ribbon states show distinct 26S proteasome accessibility, while cytoplasmic ribbons contact and deform ER‐like endomembranes.

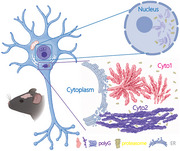

## 1 Introduction

Neuronal intranuclear inclusion disease (NIID) is defined by eosinophilic intranuclear inclusions that are ubiquitin‐ and p62‐positive and found across a broad range of tissues [1, 2, 3, 4]. Clinical presentations are diverse, but the nuclear inclusion is the most consistent pathological hallmark and remains central to diagnosis. Despite its diagnostic prominence, the native ultrastructure of NIID inclusions in neurons—how they are built in three dimensions and how they physically interface with cellular systems—remains poorly resolved. In particular, it is unclear whether NIID inclusions reflect a single uniform assembly state or a spectrum of compartment‐dependent architectures.

A major genetic cause of NIID, particularly in East Asian populations, is an abnormal CGG repeat expansion in the 5′ untranslated region of NOTCH2NLC [2, 3, 4]. Rather than silencing NOTCH2NLC, this expansion is linked to toxic gain‐of‐function mechanisms. Non‐canonical translation of the expanded repeat produces poly‐glycine (polyG) peptides that have been detected within NIID inclusions in patient tissues and model systems, positioning polyG as a key molecular component of NIID pathology [5, 6]. Notably, NIID represents the most prevalent polyG diseases in East Asian populations. Other polyG diseases include Fragile X–associated tremor/ataxia syndrome (FXTAS) [7], oculopharyngodistal myopathy (OPDM) [8, 9], and spinocerebellar ataxia type 4 (SCA4) [10]. Although the pathogenic polyG proteins in these disorders arise from distinct genetic loci, they converge on shared neuropathological and molecular mechanisms [11, 12]. This mechanistic convergence strongly implicates the polyG peptide itself—as a conserved, aggregation‐prone, and intrinsically disordered core motif—as a plausible contributor to shared pathogenic processes. What remains unclear is whether polyG assembles into a single stereotyped structure or whether it can adopt multiple inclusion states in neurons. Thus, defining how polyG builds the intranuclear inclusion is essential for linking the genetic lesion to the diagnostic pathology.

Diseases associated with protein aggregation are often discussed in the context of amyloid fibrils, yet an emerging theme across neurodegenerative inclusions is architectural diversity [13, 14, 15, 16, 17, 18]. Cellular inclusions can take non‐fibrillar forms, and a single protein can populate multiple structurally distinct assemblies—structural polymorphism—that may differ in the toxic role as a gain‐of‐function [19, 20]. Inclusion architecture can regulate accessibility to proteostasis machinery and shape interactions with endomembranes, giving structural heterogeneity direct functional implications. Moreover, because inclusions occur in distinct cellular compartments, a key question is whether polyG adopts compartment‐specific architectures with different cellular interactions.

Cryo‐electron tomography (cryo‐ET) provides a direct route to resolve inclusions in their native cellular context without chemical fixation, staining, or extraction [21]. Here, we combine cryo‐ET with correlative light–electron microscopy and focused ion beam milling to determine the ultrastructure of polyG inclusions in primary cortical neurons, and we extend the approach to brain tissue from an NIID mouse model to evaluate its in vivo conservation under native physiological constraints. Using this integrated strategy, we define the native organization of polyG assemblies and uncover evidence that NIID inclusions comprise multiple structural states across cellular compartments.

## 2 Results

### 2.1 Nuclear polyG Forms Proteasome‐Rich Ribbon Networks in Neurons

To resolve the native ultrastructure of NIID‐relevant inclusions in neurons, we transduced primary cortical neurons with a GFP‐tagged construct expressing uNOTCH2NLC‐(polyG)_100_‐GFP. Neurons were transduced at DIV5 and allowed to express the protein for 8 days (DIV5 + 8). Expression of polyG produced GFP‐positive inclusions in both the cytoplasm and nucleus, with characteristic diameters of approximately 3 µm (Figure S1). The cultures were then vitrified for tomographic imaging.

Guided by the GFP signal, cryo‐electron tomography of focused ion beam‐milled neuronal lamellae revealed prominent nuclear polyG inclusions (Figure S2). The polyG inclusion cross‐sections were typically 2–3 µm in diameter and occurred as single or multiple assemblies per nucleus(Figure S2d). Their intranuclear positions varied, with inclusions observed both adjacent to the nuclear envelope and within the nuclear interior, without an obvious positional bias, indicating that nuclear inclusion formation is not restricted to a specific subnuclear site (Figure S1).

**FIGURE 1:**
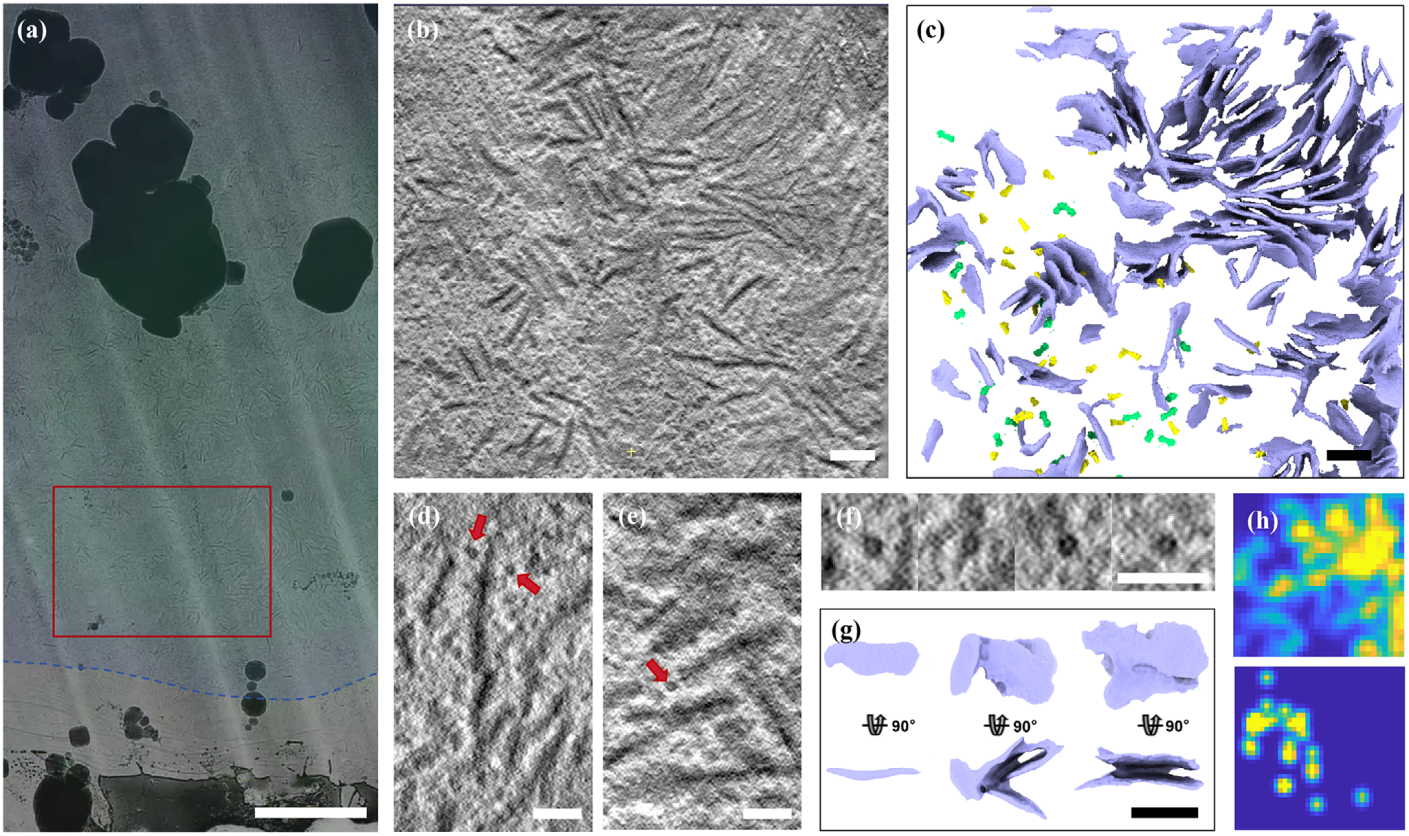
CLEM‐guided cryo‐ET reveals ribbon networks in neuronal nuclear polyG inclusions. (a) Correlative cryo‐fluorescence and low‐magnification cryo‐TEM images of a primary cortical neuron nucleus containing a GFP‐positive polyG inclusion. The nuclear area is outlined (blue). Scale bar, 1 µm. (b) Representative tomographic slice from the region indicated by the red box in (a). Scale bar, 100 nm. (c) 3D rendering of the volume shown in (b), with the nuclear polyG inclusion segmented in indigo, single‐capped 26S proteasomes in yellow, and double‐capped 26S proteasomes in green. Scale bar, 100 nm. (d, e) Higher‐magnification tomographic slices highlighting the ribbon‐like architecture of the inclusion. Red arrows indicate proteasomes. Scale bars: 50 nm. (f) Representative tomographic cross‐sections of 26S proteasomes in proximity to the inclusion. Scale bar, 50 nm. (g) Segmented ribbon network rendered from multiple viewing angles. Scale bars, 50 nm. (h) Heat maps showing the spatial distribution of the inclusion (top) and proteasomes (bottom) within the reconstructed volume shown in (b).

Three‐dimensional reconstruction and segmentation showed that nuclear inclusions comprise interconnected ribbon‐like assemblies organized into a dense network (Figure 1). Inclusions typically displayed a dense central region with tightly packed ribbons surrounded by a less dense peripheral zone (Figure 1b). Individual ribbons were elongated and flattened, with nanometer‐scale thickness and tens‐of‐nanometers width, extending for tens to hundreds of nanometers (Figure 1c). Ribbon elements frequently branched and merged, forming a 3D network rather than stacked sheets (Figure 1c,g). Notably, we did not detect periodic fibrillar features commonly observed in canonical amyloid assemblies. This ribbon morphology is in contrast to the uniform fibrils forming polyQ‐expanded huntingtin exon 1 aggregates in mammalian cells but reminiscent of ribbon‐like inclusions reported for ALS/FTD related C9ORF72‐poly‐GA inclusions in primary neurons [15, 16].

Within nuclear inclusions, we frequently observed ∼10 nm ring‐shaped particles in tomographic slices (Figure 1d–f). Template matching followed by subtomogram averaging confirmed these particles as 26S proteasomes, including both single‐ and double‐capped forms, and revealed ground‐state and substrate‐processing conformations (Figure S3) [15]. Proteasomes were strongly enriched inside nuclear inclusions compared with the surrounding nucleoplasm, reaching 600 nM inside inclusions vs. 40 nM in nucleoplasm (Table S1). These measurements indicate that the nuclear ribbon network concentrates 26S proteasomes. Proteasome association has been reported for several inclusion types, including tau, TDP‐43, and poly‐GA, although the enrichment reported in those contexts appears much stronger [13, 15, 17].

### 2.2 Cyto1 and Cyto2 Define Cytoplasmic Ribbon Polymorphs With Distinct Proteasome Access

**FIGURE 2:**
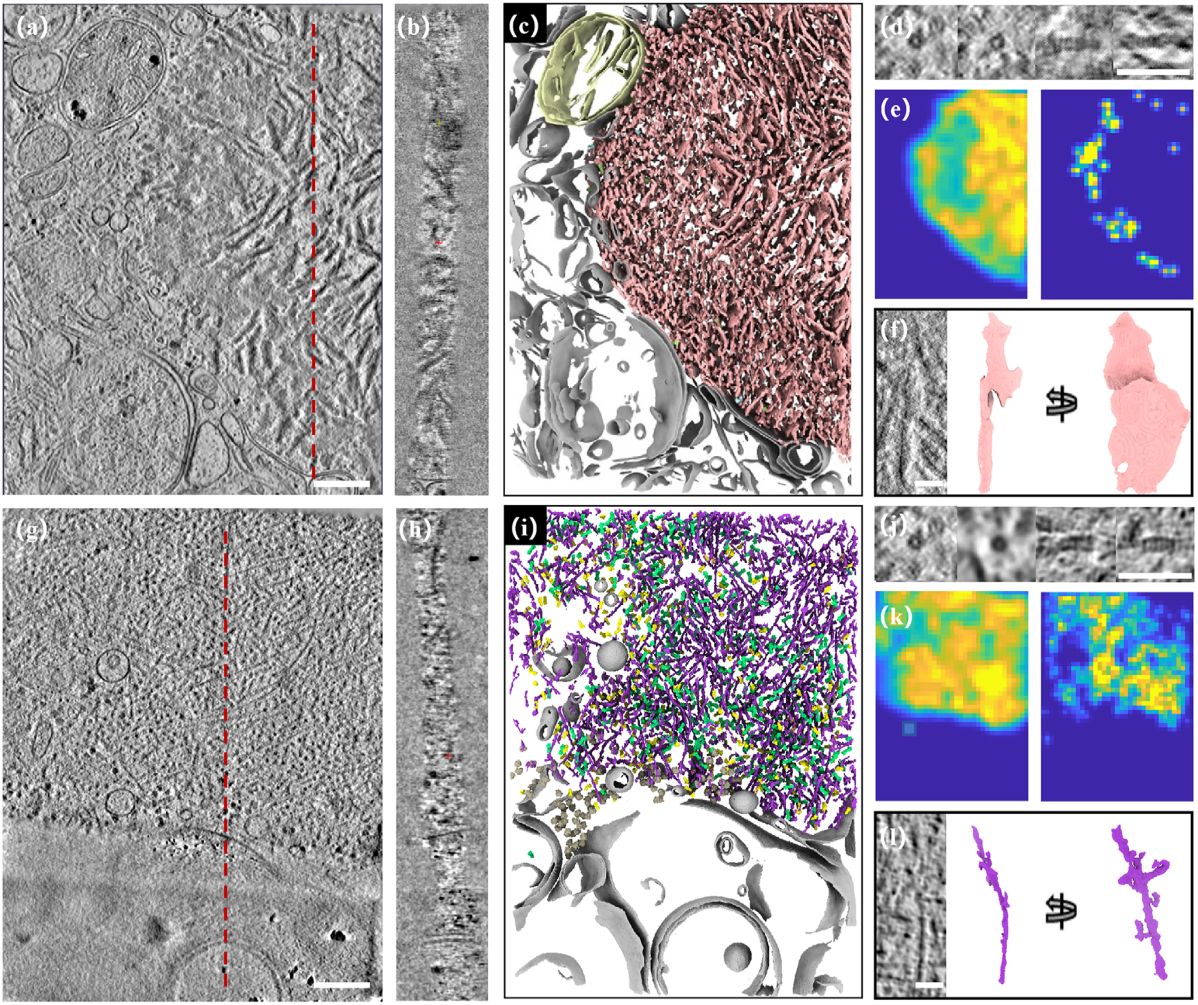
Polymorphic cytoplasmic inclusions in primary neurons. (a, b) Representative slice‐and‐view visualization of a tomogram containing a Cyto1 inclusion. (a) Central tomographic slice showing the overall organization of the inclusion. (b) Orthogonal y–z view corresponding to the region indicated by the red line in (a). Scale bar, 200 nm. (c) 3D rendering of the tomogram in (a). The Cyto1 inclusion is shown in pink, mitochondria in light yellow, the endomembrane system in gray, single‐capped 26S proteasomes in yellow, and double‐capped 26S proteasomes in green. (d) Higher‐magnification tomographic slice highlighting proteasomes in the vicinity of the Cyto1 inclusion. Scale bar, 50 nm. (e) Heat maps showing the spatial distribution of the Cyto1 inclusion (left) and proteasomes (right) within the reconstructed volume. (f) Higher‐magnification tomographic slice and corresponding 3D rendering highlighting the Cyto1 ribbon network. Scale bar, 50 nm. (g–l) Corresponding analyses for a Cyto2‐type cytoplasmic inclusion. (g, h) Representative slice‐and‐view visualization of a tomogram containing a Cyto2 inclusion. (g) Central tomographic slice showing the overall organization of the inclusion. (h) Orthogonal y–z view corresponding to the region indicated by the red line in (g). Scale bar, 200 nm. (i) 3D rendering with the Cyto2 inclusion shown in purple. (j) Higher‐magnification tomographic slice. Scale bar, 50 nm. (k) Heat maps showing spatial distributions of the Cyto2 inclusion (left) and proteasomes (right). (l) Higher‐magnification tomographic slice with corresponding 3D rendering. Scale bar, 50 nm. Colors for proteasomes and endomembranes are as in (b).

In addition to nuclear inclusions, primary neurons contained abundant cytoplasmic polyG inclusions. Cryo‐ET guided by the GFP signal resolved two cytoplasmic inclusion types, termed Cyto1 and Cyto2, distinguished by their ribbon organization and subcellular distribution (Figure 2). Cyto1 inclusions were composed of wider, tightly packed ribbons forming compact networks and were frequently located near the nuclear envelope (Figure 2a–f). In contrast, Cyto2 inclusions comprised thinner, more fragmented ribbons arranged in looser, more porous networks and were more often observed away from the nucleus or near neuronal processes (Figure 2g–l). Although both types typically measured ∼1–3 µm in diameter, their internal architectures were clearly distinct, indicating that polyG does not adopt a single uniform cytoplasmic organization (Figure 2f,l).

Consistent with the strong proteasome enrichment observed in nuclear ribbon inclusions, proteasome‐like particles were also readily detected near cytoplasmic inclusions (Figure 2d,j). Template matching and subtomogram averaging identified these particles as 26S proteasomes. However, in the cytoplasm, their distribution depended on inclusion type. In Cyto2 inclusions, proteasomes were enriched throughout the ribbon network interior, reaching an overall concentration of 1370 nM (Figure 2k; Table S2). In contrast, Cyto1 inclusions largely excluded proteasomes from densely packed cores, with a limited number of proteasomes identified predominantly at the periphery (Figure 2e). These observations indicate that the cytoplasmic ribbon packing state modulates access of the proteostasis machinery to the inclusion interior.

To independently validate the cryo‐ET observation of state‐dependent proteasome association, we performed immunofluorescence microscopy using antibodies against proteasome subunits. Cytoplasmic inclusions showed substantial but incomplete colocalization with proteasome signals (Figure S4). Quantification indicated that approximately 70% of cytoplasmic inclusions colocalized with proteasomes, whereas a substantial fraction did not (Figure S4b,c). This partial colocalization is consistent with the inclusion‐type heterogeneity observed by cryo‐ET, particularly the contrasting proteasome accessibility of Cyto1 and Cyto2 states.

### 2.3 Cytoplasmic Ribbon Polymorphs Interact With Endomembranes and Induce Membrane Deformation

**FIGURE 3:**
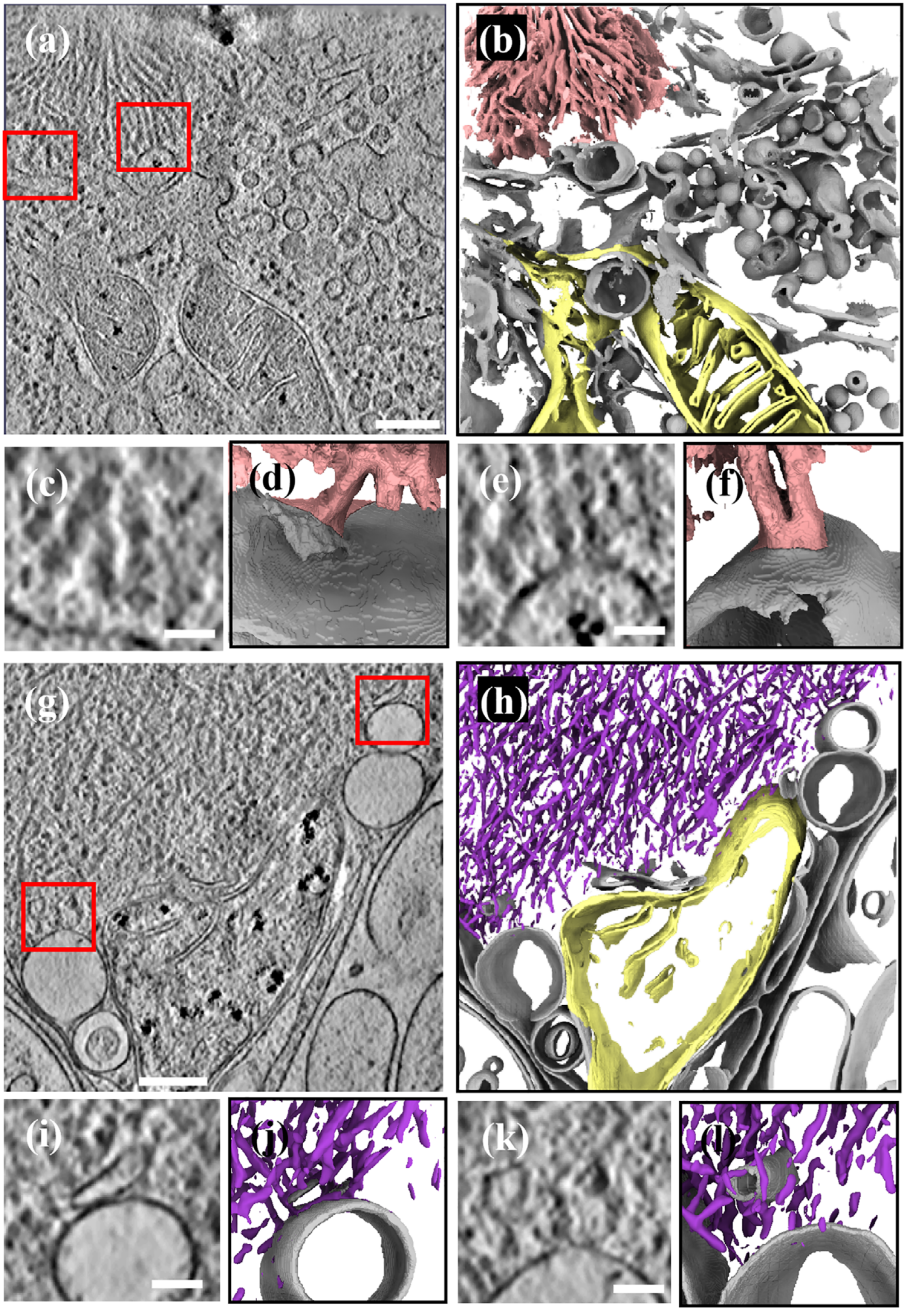
Cytoplasmic ribbon polymorphs engage endomembranes and deform ER‐like membranes. (a, g) Representative tomographic slices of Cyto1‐ (a) and Cyto2‐type (g) cytoplasmic inclusions. Scale bars, 200 nm. (b, h) 3D renderings corresponding to (a, g). Cyto1 inclusion is shown in pink, Cyto2 inclusion in purple, mitochondria in yellow, and the endomembrane system in gray. Scale bars, 200 nm. (c–f, i–l) Higher‐magnification tomographic slices (c, e, i, k) and corresponding 3D renderings (d, f, j, l) highlighting close contacts between ribbon inclusions and endomembranes. Ribbon–membrane interfaces are accompanied by membrane bending and local deformation at contact sites. Scale bars, 50 nm.

Both cytoplasmic ribbon inclusion types frequently engaged cellular endomembranes, most prominently endoplasmic reticulum (ER)–like membranes, but also vesicles and occasional double‐membrane structures (Figures 2 and 3). Direct ribbon–membrane contact sites were evident, and these interactions were accompanied by membrane bending, local deformation, and occasional disruption at the interface (Figure 3). Thus, despite their distinct ribbon packing states, both Cyto1 and Cyto2 inclusions share a capacity to engage endomembrane systems and physically perturb membrane architecture, reminiscent of membrane‐associated phenotypes reported for polyQ inclusions [16]. In contrast, membrane associations were not detected within nuclear inclusions, consistent with compartment‐specific environments.

**FIGURE 4:**
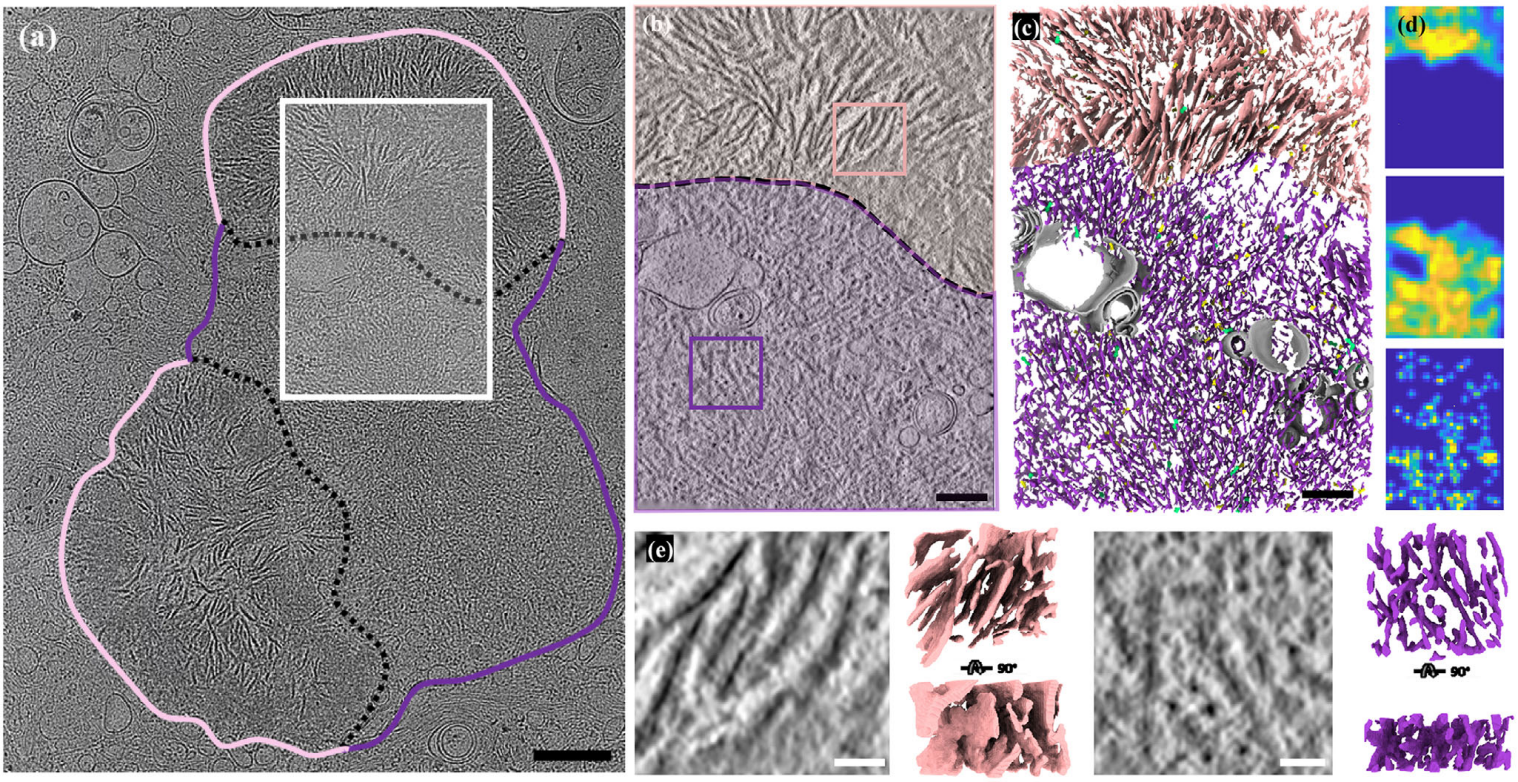
Cyto1 and Cyto2 cytoplasmic ribbon states can coexist and spatially interact within single neurons. (a) Low‐magnification cryo‐TEM image showing a neuron containing both Cyto1‐ and Cyto2‐type cytoplasmic inclusions. Colors indicate Cyto1 (pink) and Cyto2 (purple). Scale bar, 500 nm. (b) Representative tomographic slice of the white boxed region in (a) containing both inclusion types. Scale bar, 200 nm. (c) 3D rendering of the volume in (b), with Cyto1 (pink), Cyto2 (purple), single‐capped 26S proteasomes (yellow), double‐capped 26S proteasomes (green), and endomembranes (gray). Scale bar, 200 nm. (d) Heat maps showing the spatial distribution of Cyto1 (top), Cyto2 (middle), and proteasomes (bottom) within the reconstructed volume. (e) High‐magnification 3D rendering of the boxed regions in (b) of both inclusion types. Scale bar: 50 nm. (f) Volumetric fraction of ribbon‐like assemblies within Cyto1 and Cyto2 inclusions (*n* = 8 tomograms per group). Data are mean ± s.d.; ^****^ *p* < 0.0001 (unpaired two‐tailed Student's t‐test). (g) Total 26S proteasome concentration within Cyto1 and Cyto2 inclusions (*n* = 8 tomograms per group). Data are mean ± s.d.; ^****^ *p* < 0.0001 (unpaired two‐tailed Student's t‐test).

Unexpectedly, Cyto1 and Cyto2 inclusions could be detected within the same neuron, indicating coexistence of distinct cytoplasmic ribbon states within a shared cellular environment. In some cases, Cyto1‐ and Cyto2‐like architectures were closely apposed, with a Cyto2‐like core surrounded by a Cyto1‐like region (Figure 4). In these core–shell assemblies, the Cyto2‐like core was more proteasome‐rich than the surrounding Cyto1‐like shell, consistent with the distinct proteasome accessibility of the two ribbon states (Figure 4d). This spatial organization suggests that different packing states can coexist within a continuous inclusion‐associated compartment and may be arranged in a layered manner. While the functional significance of this core–shell organization remains unclear, the close apposition of Cyto1‐ and Cyto2‐like architectures raises the possibility that cytoplasmic ribbon states may be related through remodeling or interconversion. However, based on the current structural and localization data, we cannot infer directionality, maturation order, or relative cytotoxicity of the two states.

### 2.4 PolyG Polymorphism is Conserved In Vivo

To determine whether the inclusion polymorphism observed in cultured neurons is preserved in vivo, we performed cryo–electron tomography on brain tissue from an NIID mouse model expressing expanded polyG without any tag that mimics the multisystemic impairments associated with polyG inclusions [22]. This model recapitulates key NIID‐associated phenotypes, including polyG‐positive, ubiquitin‐ and p62‐positive inclusions, neurodegenerative changes, and motor and cognitive deficits [22].

**FIGURE 5:**
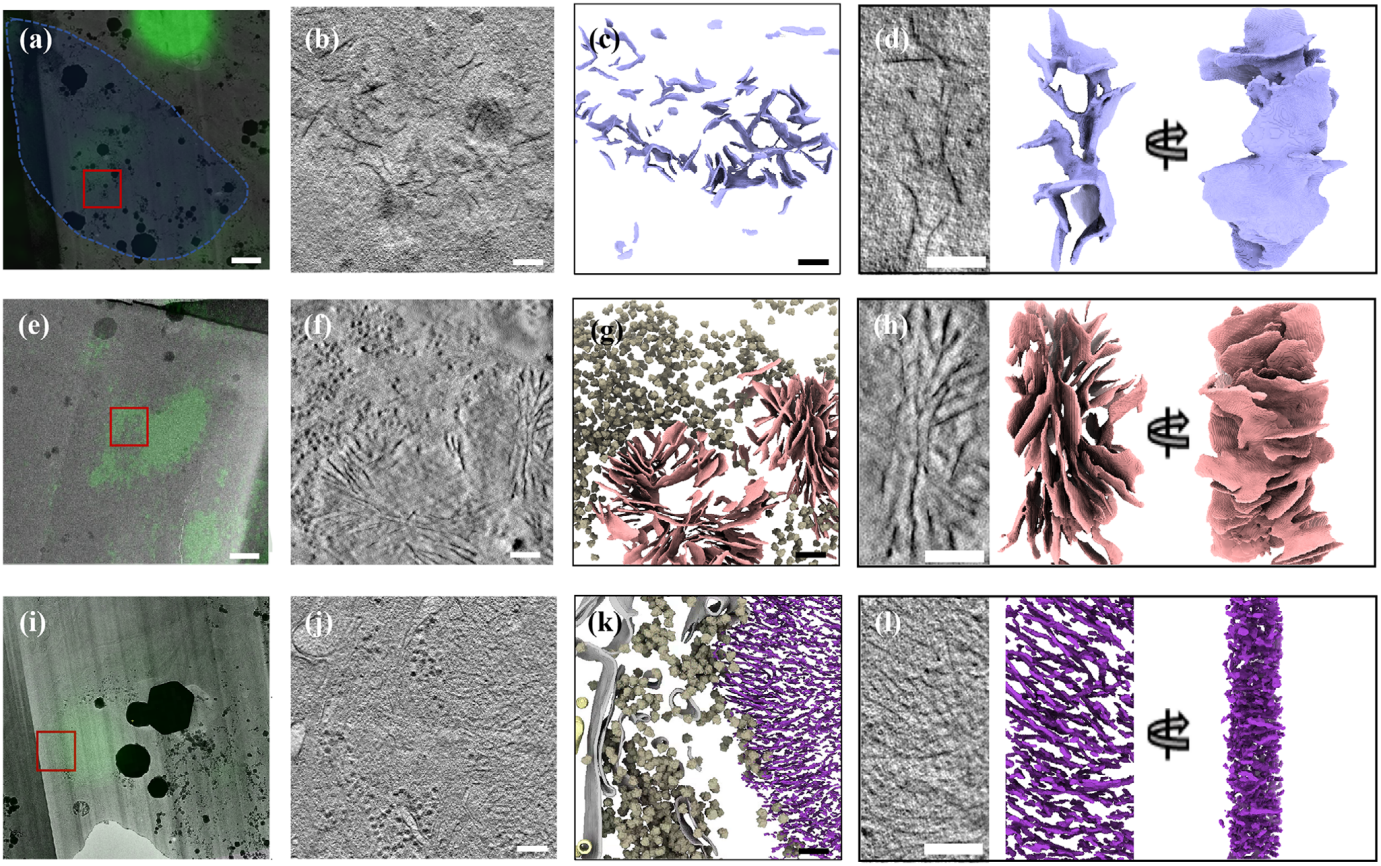
PolyG inclusion polymorphism is conserved in vivo in mouse brain tissue. (a) Correlative cryo‐fluorescence and low‐magnification cryo‐TEM images of a neuron in hippocampal tissue containing an intranuclear inclusion. The nucleus is outlined (blue). Scale bar, 1 µm. (b–d) Representative views of the intranuclear inclusion in tissue: (b) tomographic slice. Scale bar, 100 nm. (c) 3D rendering of the reconstructed volume, with the intranuclear inclusion shown in blue. (d) Higher‐magnification tomographic view highlighting ribbon‐like assemblies within the inclusion. Scale bar, 20 nm. (e–h) Corresponding analyses of a Cyto1‐like cytoplasmic inclusion in tissue: (e) low‐magnification cryo‐TEM image. Scale bar, 1 µm. (f) Tomographic slice. Scale bar, 100 nm. (g) 3D rendering with the Cyto1‐like inclusion shown in pink and ribosomes in yellow. (h) Higher‐magnification tomographic view highlighting ribbon‐like assemblies. Scale bar, 20 nm. (i–l) Corresponding analyses of a Cyto2‐like cytoplasmic inclusion in tissue: (i) low‐magnification cryo‐EM image. Scale bar, 1 µm. (j) tomographic slice. Scale bar, 100 nm. (k) 3D rendering with the Cyto2‐like inclusion shown in purple, ribosomes in yellow, and endomembranes in gray. (l) Higher‐magnification tomographic view highlighting ribbon‐like assemblies. Scale bar, 20 nm.

Given that polyG inclusions frequently colocalized with p62‐positive aggregates in the brain tissues of the NIID mouse model (Figure S5), we administered p62–eGFP AAV via stereotactic hippocampal injection into 8‐week‐old mice. Animals were sacrificed and tissues harvested three weeks post‐injection to enable high‐resolution spatial mapping and precise subcellular localization of polyG inclusions in situ. Brain samples were processed using the tissue cryo‐ET workflow developed in‐house [23] (Figure S6). The tissue was well vitrified, as verified by tomographic reconstructions showing preserved cellular ultrastructure, including intact synapses (Figure S7a–c). This p62‐guided, CLEM‐based approach enabled robust identification of both nuclear and cytoplasmic inclusions in hippocampal neurons (Figure S7; Figure 5).

Nuclear inclusions in brain tissue displayed a ribbon‐network architecture closely resembling that observed in primary neurons, with densely interconnected ribbons forming compact intranuclear assemblies and no detectable membrane association (Figure 5a–d). Cytoplasmic inclusions similarly segregated into Cyto1‐ and Cyto2‐like ribbon architectures, supporting conservation of cytoplasmic multiple ribbon states in vivo (Figure 5e–l). In addition, ribbon‐containing inclusions were observed within neuronal processes, including dendrites, revealing an additional spatial manifestation of polyG ribbon assemblies in the intact brain (Figure S7d–f). Together, these observations indicate that a multi‐form inclusion repertoire is observed in both systems (Figure S8).

Consistent with the findings in primary neurons, polyG inclusions exhibiting colocalization with proteasomal subunits were also detected in brain sections from the NIID mouse model (Figure S9). To further assess the cellular consequences of polyG accumulation, we analyzed RNA‐seq data from the NIID mouse brains. Differential expression analysis identified 1203 genes, including 702 upregulated (58.4%) and 501 downregulated (41.6%) transcripts. Functional annotation revealed enrichment of genes associated with proteasome‐related processes (94 genes) and ER‐related processes (75 genes). Notably, genes involved in ER stress response pathways were significantly upregulated (Figure S10). These transcriptomic alterations are consistent with the cryo‐ET observations of proteasome recruitment and extensive interactions between polyG inclusions and membrane‐associated structures, suggesting that polyG accumulation is accompanied by disturbances in proteostasis and ER‐associated cellular stress pathways.

## 3 Discussion

Classical EM described NIID‐associated polyG inclusions as filamentous networks [24, 25]. Our in situ cryo‐ET reframes this picture by showing that polyG assemblies adopt a ribbon‐based architecture, aligning NIID polyG with other glycine‐rich inclusion contexts such as cellular poly‐GA [15]. This architectural class may help explain why polyG inclusions have remained difficult targets for the types of high‐resolution structural pipelines that have been successful for canonical amyloid fibrils, including tau, Aβ, and α‐synuclein [19, 26, 27, 28]. Importantly, the ribbon organization is observed in both neuronal culture and brain tissue, supporting that it reflects an intrinsic assembly mode rather than a tag‐driven artifact. Consistent with our in situ ultrastructural observations, Congo red staining, a classical histological marker of amyloid deposits, failed to detect polyG inclusions in NIID patient tissue (Figure S11). These dye‐staining results further support the notion that polyG assemblies differ from conventional amyloid fibrils [29, 30].

A key implication of our data is that polyG populates multiple structural states in situ, and that these states are shaped by subcellular compartment. Interestingly, biophysical studies have shown that polyglycine can adopt multiple conformational states, including β‐sheet‐like and alternative packing arrangements [31, 32, 33, 34, 35]. Although our cryo‐ET data do not resolve the underlying molecular fold, the distinct ribbon architectures observed here suggest that polyG assemblies can exist in multiple higher‐order organizational states, consistent with the intrinsic structural plasticity of polyglycine. Structural polymorphism—often discussed in the context of prion‐like strains—has been documented for several neurodegeneration‐related proteins and has been linked to biological heterogeneity across diseases and even within individuals [36, 37, 38]. Our results extend this paradigm to NIID‐relevant polyG and suggest that “strain‐like” diversity can emerge not only as globally distinct inclusion types, but also within a single cell. The observation of Cyto1 and Cyto2 states within the same neuron, and their occasional core–shell organization, is consistent with state coexistence and raises the possibility of remodeling or interconversion. However, the transition between Cyto1 and Cyto2 would likely involve substantial structural remodeling, as suggested by their distinct properties, including the differential enrichment of proteasomes. At the same time, the present data do not establish temporal order or directionality, and therefore do not support claims about maturation stage or relative cytotoxicity of specific states.

The most mechanistically actionable axis revealed here is an architecture‐to‐accessibility relationship for proteostasis machinery. Proteasomes are a recurring feature of inclusion biology across multiple disorders [13, 14, 15, 39, 40], but our data indicate that polyG inclusions differ not only in whether proteasomes are present, but in how deeply proteasomes can access the inclusion interior. The sharp contrast between proteasome penetration of Cyto2 vs. exclusion from dense Cyto1 cores suggests that inclusion packing may gate entry of large macromolecular complexes. We do not directly measure proteasome activity and therefore avoid equating enrichment with inhibition, while our previous work shows such enrichment is the consequence of inhibition [13, 15]. Notably, proteasome enrichment around polyG inclusions appears weaker than that reported for several other aggregate systems [13, 15, 17]; however, the basis for this difference remains unclear and warrants further investigation.

In parallel, cytoplasmic polyG assemblies display robust engagement with endomembranes, particularly ER‐like membranes, with direct contacts accompanied by membrane deformation. This points to a second interaction axis—inclusion–endomembrane coupling—that is selectively available in the cytoplasm and absent from the nuclear compartment. RNA‐seq signatures indicative of upregulated ER stress response pathways provide convergent molecular evidence supporting disruption of ER homeostasis in the context of polyG expression (Figure S10). Although causality cannot be inferred from morphology alone, the combination of membrane deformation and ER‐stress signatures motivates direct tests of whether inclusion–ER contacts alter ER trafficking, or local proteostasis at the ER interface [16, 41].

Extending cryo‐ET from cultured neurons to brain tissue strengthens the physiological relevance of these conclusions: the major inclusion states observed in culture are conserved in vivo, and tissue anchors inclusion architecture within intact neuronal geometry, including neuritic compartments. Notably, expressing the same construct in U2OS cells yields predominantly a single cytoplasmic inclusion morphology (Figure S8), underscoring that accessible inclusion states are constrained by cellular context. Together, these comparisons argue that NIID‐relevant inclusion diversity is best captured in neurons and validated in tissue, where compartmentalization, membrane organization, and proteostasis networks more closely reflect disease‐relevant environments. In summary, our work places NIID polyG inclusions within a broader framework of non‐canonical, structurally polymorphic assemblies and identifies two interaction axes—proteasome accessibility and endomembrane coupling—that vary across inclusion states and compartments, providing an in situ structural foundation for mechanistic experiments linking inclusion architecture to proteostasis regulation, membrane homeostasis, and neuronal vulnerability in NIID.

## 4 Experimental Section/Methods

### 4.1 Animals

Mouse models expressing expanded uN2CpolyG protein were generated as previously reported [22]. All animal housing and use procedures were approved by the Institutional Animal Care and Use Committees of Peking University (Approval Number: LSC‐GuoQ‐1).

### 4.2 Primary Rat Cortical Neuron Culture

Primary neuronal cultures were prepared using a previously established protocol [15]. Briefly, brains from neonatal Sprague–Dawley rats (< 24 h old) were rapidly isolated after a T‐shaped cranial incision and transferred into dissection medium consisting of DMEM/F12 supplemented with 1% penicillin–streptomycin. Cerebral cortices were dissected, and meninges and blood vessels were carefully removed. The tissue was minced and digested with 0.25% trypsin (Thermo Fisher Scientific) at 37°C for 15 min with gentle agitation. Digestion was terminated by adding resuspension medium. The tissue was then gently triturated until no large clumps remained, allowed to settle for 3 min, and the supernatant was collected and centrifuged at 500 × g for 2 min. The cell pellet was gently resuspended by pipetting approximately 10–12 times to obtain a single‐cell suspension.

Cells were plated at a density of 1.0 × 10^6^ cells mL^−1^ onto poly‐D‐lysine‐coated dishes and maintained at 37°C in a humidified incubator with 5% CO_2_. After 2 h to allow cell attachment, the medium was replaced with neuron maintenance medium consisting of Neurobasal medium (Thermo Fisher Scientific) supplemented with 2% B27, 1% GlutaMAX, and 1% penicillin–streptomycin, together with 10 µM 5‐fluoro‐2′‐deoxyuridine. After 48 h, the medium was replaced with standard neuron maintenance medium without 5‐fluoro‐2′‐deoxyuridine.

Adenoviral vectors expressing uNOTCH2NLC‐(polyG)_100_‐GFP or GFP alone were generated using the AdMax system (Vigene Biosciences), as described previously [15]. The expression cassette was driven by the CMV promoter. Viral particles were produced and titered by Vigene Biosciences (Jinan, China), and the viral titer used for transduction was 4.7 × 10^10^ PFU mL^−1^. On day 4 post‐plating, when clear neuronal morphology was observed by light microscopy, half of the culture medium was replaced with neuron maintenance medium containing adenoviral particles encoding uNOTCH2NLC‐(polyG)_100_‐GFP or GFP alone at approximately 0.5 µL virus per mL medium. Twenty‐four hours after infection, the medium was fully replaced with fresh neuron maintenance medium, followed by half‐medium changes every 2–3 days. Cells were processed for sample preparation 7–8 days after viral infection.

### 4.3 Cryo‐ET Sample Preparation for Cortical Neuron Culture

For neuron culture, 200‐mesh gold EM grids (R2/1 gold, Quantifoil Micro Tools, Germany) were glow‐discharged and UV‐irradiated. Grids were transferred to 35‐mm glass‐bottom dishes and fully immersed in poly‐D‐lysine (PDL, 1 mg mL^−1^). After incubation at 37°C and 5% CO_2_ for 24 h, grids were rinsed three times with deionized water and incubated in resuspension medium (DMEM/F12, Thermo Fisher Scientific; supplemented with 10% fetal bovine serum (FBS) and 1% penicillin–streptomycin). Cryo‐ET samples were prepared 8 days post‐infection by plunge‐freezing into a mixture of liquid ethane and propane (ethane:propane = 36.9%:63.1%) using a Vitrobot Mark IV (Thermo Scientific) at a chamber temperature of 37°C and 0% humidity. The vitrified grids were stored in liquid nitrogen until cryo‐CLEM imaging and cryo‐FIB milling.

### 4.4 Cryo‐CLEM and Cryo‐FIB Milling of Neuronal Samples

Vitrified neuronal grids were clipped into Autogrids (ThermoFisher Scientific) before being loaded onto the cryo‐stage of the Thunder Imager EM Cryo‐CLEM (Leica) equipped with a 50×/0.9 NA objective at liquid‐nitrogen temperature. Whole‐grid fluorescence and bright‐field montages were acquired and stitched. For sample thinning, grids were transferred to an Aquilos 2 Cryo‐FIB/SEM (Thermo Fisher Scientific) and milled with a protocol established before [42]. In brief, a thin organometallic platinum layer was deposited using the gas injection system (GIS) to improve conductivity, and SEM overview images were acquired. Fluorescence/bright‐field coordinates were correlated to SEM space using three‐point correlation in MAPS software, enabling identification of the target region. The stage was tilted to achieve a 12° incidence geometry between the ion beam and the grid plane. Coarse milling used a 30 kV gallium beam at 0.5 nA; current was reduced stepwise, and final polishing was performed at 30 pA to generate lamellae of ∼100–200 nm thickness.

### 4.5 Immunofluorescence

Immunostaining was performed 8 days post‐infection. Cells were washed three times with pre‐chilled PBS and fixed with freshly prepared 4% paraformaldehyde for 30 min at room temperature. After three PBS washes, cells were permeabilized with 0.2% Triton X‐100 in PBS for 15 min at 4°C, then washed three times with PBST (PBS + 0.1% Tween‐20). Cells were blocked in 5% BSA in PBST for 1 h at room temperature and incubated overnight at 4°C with primary antibody (rabbit anti‐PSMC4 antibody, Proteintech 11389‐1‐AP, 1:250; mouse anti‐polyG antibody, 4D12 [5], 1:100; rabbit anti‐p62 antibody, Proteintech 18420‐1‐AP), and second antibody (Alexa Fluor Plus 555–conjugated goat anti‐rabbit IgG secondary antibody, A32732; 1:500; Thermo Fisher Scientific). Images were taken using laser‐scanning confocal microscopy (Nikon A1MP).

### 4.6 Recombinant AAV Vector Construction and Virus Production

Total RNA was isolated from mouse hippocampal neurons and reverse‐transcribed into cDNA, which served as the template for PCR amplification of the coding sequence of mouse p62 (SQSTM1). The GenBank accession number for the target sequence is NM_011018.3. The amplified fragment was cloned into an AAV vector backbone carrying an enhanced green fluorescent protein (EGFP) reporter gene driven by the human synapsin promoter (hSyn) using homologous recombination, generating the recombinant p62‐EGFP fusion expression vector. All subsequent steps, including AAV vector construction, AAV9 serotype virus packaging, and purification, were performed by WZ Biosciences Inc. The titer of the p62‐EGFP‐AAV9 virus used in this study was determined to be 9.66 × 10^1^ ^3^ vg/mL (viral genomes per milliliter). The virus was aliquoted and stored at −80°C in the dark to avoid repeated freeze‐thaw cycles.

### 4.7 Stereotaxic Injection into the Mouse Hippocampal Dentate Gyrus

Two‐month‐old neuronal intranuclear inclusion disease (NIID) model mice were deeply anesthetized via intraperitoneal injection of 1% pentobarbital sodium (50 mg/kg) and secured in a stereotaxic frame. The scalp was shaved and disinfected, a 1‐cm midline incision was made over the calvarium, and the subcutaneous tissue was bluntly dissected to expose the skull. The level of the bregma was adjusted to eliminate head tilt. Coordinates for bilateral injection into the hippocampal dentate gyrus (DG) were defined based on the Mouse Brain Stereotaxic Atlas, relative to bregma: anteroposterior (AP) −2.0 mm, mediolateral (ML) ±1.3 mm, dorsoventral (DV) −2.0 mm from the dura mater. Coordinates were adjusted slightly for individual mice according to skull thickness prior to injection. Using a microinjection pump, 0.5 µL of p62‐EGFP‐AAV9 virus solution was infused into each DG at a constant rate of 0.1 µL/min, corresponding to a total dose of approximately 4.83 × 10^10^ viral genomes (vg) per side. Following injection, the needle was left in place for 5 min to prevent virus reflux, then slowly withdrawn. The burr holes were sealed with bone wax, the skin incision was sutured, and the surgical site was disinfected again. Postoperatively, mice were placed on a 37°C heating pad for recovery. After full awakening, they were returned to specific pathogen‐free (SPF) housing with ad libitum access to food and water. Mice were processed for frozen tissue sample preparation 21 days post‐injection to ensure stable expression of the p62‐EGFP fusion protein in hippocampal neurons.

### 4.8 High‐Pressure Freezing of Tissue Samples

NIID model mice were anesthetized with isoflurane. The thoracic cavity was opened, a needle was inserted into the left ventricle, and the right atrium was incised. Ice‐cold NMDG–HEPES buffer was perfused at ∼10 mL min^−1^ until peripheral tissues blanched. The procedure was completed within 10 min to minimize hypoxic injury; buffers were kept cold throughout.

Brains were extracted into ice‐cold NMDG–HEPES buffer. The cerebellum was removed and the caudal end trimmed flat. Brains were mounted on a Leica VT1200S vibratome and sectioned (200 µm) at 0.26 mm s^−1^ under ice‐cold buffer immersion. Hippocampal regions were isolated using a tissue punch, transferred to NMDG–HEPES buffer, and manually trimmed to ∼0–150 µm. Tissue blocks were stained with Hoechst for 10 min, then incubated in NMDG–HEPES supplemented with 20% (w/v) dextran and 5% (w/v) sucrose prior to cryo‐sample preparation.

The tissue sample vitrification was established as described before [43]. EM grids were glow‐discharged prior to tissue mounting. Tissue blocks were transferred onto the carbon side of the EM grid; excess liquid was blotted from the opposite side. Grids with tissue were loaded into a carrier filled with 2‐methylpentane, capped with a sapphire disc (avoiding air bubbles), and high‐pressure frozen. Carriers were stored in liquid nitrogen. For downstream cryo‐FIB preparation, the 2‐methylpentane was removed at cryogenic temperature by transferring carriers into a −170°C liquid ethane/propane mixture, releasing grids for subsequent FIB operations.

### 4.9 Cryo‐CLEM and Lift‐Out of Brain Tissue Samples

Tissue lift‐out was performed on a Helios 5 Cryo‐FIB/SEM (Thermo Fisher Scientific) equipped with an integrated light microscope, using a protocol established before [23]. A panoramic SEM image was acquired to locate tissue blocks. The tissue surface was flattened to create a plane for fluorescence imaging, followed by z‐stack acquisition in iFLM (Hoechst and EGFP channels). Fluorescence and ion‐beam images were correlated in MAPS to localize inclusion‐rich regions.

A 40 × 30 µm block was isolated by ion‐beam milling, polished, and attached to a lift‐out needle via redeposition. The block was transferred to a rectangular‐hole support grid, anchored bilaterally, and sectioned to produce ∼3 µm thick slabs. Final lamella thinning followed the same strategy as neuronal lamellae, with integrated fluorescence monitoring to retain targets.

### 4.10 Cryo‐ET Data Collection and Reconstruction

Cryo‐electron tomography tilt series were acquired on a Titan Krios (Thermo Fisher Scientific, G3) operated at 300 kV and equipped with a K3 detector (Gatan). Neuronal lamellae were collected at 26 000× magnification (3.33 Å/pixel), and tissue lamellae at 33 000× magnification (2.64 Å/pixel). The defocus range was −4 to −6 µm. Tilt series were acquired in 2° increments over −52° to +68° (starting at 12°) using the PACE‐TOMO [44]. Ten frames were recorded per tilt, resulting in a cumulative dose of 110–140 e^−^ Å^−2^ per tilt series.

Additional tissue datasets were acquired on a Titan Krios (Thermo Fisher Scientific, G4) equipped with a Gatan energy filter and a Falcon 4i direct electron detector, using Tomography 5 software for automated dose‐symmetric acquisition. Data were collected at 42 000× magnification (3.0 Å/pixel) with a tilt range of −61° to +39° (starting at −11°) in 2° increments. The defocus range was −4 to −6 µm, and an electron dose of 2 e^−^ Å^−2^ was applied per tilt.

Frame alignment/motion correction was performed with MotionCor2 [45]. Tilt series were aligned in IMOD using patch tracking and reconstructed using weighted back‐projection [46].

### 4.11 Segmentation of Inclusions and Membranes

Membranes, including vesicles, mitochondria, and nuclear membranes, were segmented using MemBrain [47]; volumes were rescaled as needed to match the model's expected pixel size. Missed membrane regions were supplemented manually in Amira (Thermo Fisher Scientific) [48].

PolyG inclusions were segmented using EMAN2 TomoSeg [49], trained per tomogram using manual annotations to maximize specificity for ribbon‐like densities. Outputs were cleaned by masking non‐target structures when needed.

Inclusion compactness/accessibility was quantified as VG/VIV_G/V_IVG/VI, where VGV_GVG is segmented ribbon volume, and VIV_IVI is total inclusion volume defined in Amira [48].

### 4.12 Proteasome Localization and Subtomogram Averaging

Proteasome‐like ring particles were identified by combined manual inspection and template matching. A rat 26S proteasome density map (EMD‐3916) was low‐pass filtered, resampled to 13.32 Å per pixel, and cropped to a 160‐pixel box using RELION [50] tools to generate a template. Template matching was performed using STOPGAP [51] to obtain particle coordinates and orientations. Matches were screened in tom_av3_stackbrowser to remove false positives [52]. Subtomograms were extracted using Warp [53]. For neuronal samples, 7431 particles were extracted from 43 tomograms; after screening, 5808 particles remained. 3D classification/refinement in RELION produced single‐capped and double‐capped 26S proteasome classes.

For 19S conformational analysis, double‐capped proteasomes were split to generate two particles, while single‐capped proteasomes yielded one particle each. The combined dataset (7270 particles) was classified into ground and substrate‐processing states based on established structural criteria [15].

### 4.13 Proteasome Concentration Analysis

Proteasome localization relative to polyG inclusions was quantified based on 3D tomographic segmentation and particle coordinates. For each tomogram, polyG inclusion regions were manually segmented in Amira to generate binary masks, in which voxels assigned to the inclusion were defined as the “inside” region and the remaining tomographic volume was defined as the “outside” region. The corresponding volumes of the inside and outside regions were calculated from the segmented voxel numbers after conversion using the calibrated voxel size of each tomogram. Proteasome particles were identified in three dimensions, and their coordinates were mapped onto the corresponding binary masks to determine whether each particle was located inside or outside the inclusion region. This procedure was applied to quantify total proteasome particles as well as single‐capped and double‐capped proteasome populations in each compartment. Proteasome concentration was calculated as C = N / (N_A_ × V), where N is the number of proteasome particles, N_A_ is Avogadro's constant, and V is the corresponding segmented volume in liters. Concentrations were converted to µM for reporting.

### 4.14 RNA Sequencing and Bioinformatic Analysis

RNA‐seq and bioinformatic analyses were carried out in collaboration with OE Biotech Co., Ltd. (Shanghai, China), following methods described previously [54]. Brain tissues from the mouse model were harvested, and total RNA was isolated from homogenized brain tissues using the EasyPure RNA Kit (TransGen Biotech, Beijing, China), with RNA integrity verified by Agilent Bioanalyzer (RIN ≥ 7.0). Libraries were constructed using TruSeq Stranded mRNA LTSample Prep Kit (Illumina) according to the manufacturer's instructions. These libraries were sequenced on the Illumina sequencing platform (HiSeqTM 2500 or Illumina Hi Seq X Ten), and 125 bp/150 bp paired‐end reads were generated. Differentially expressed genes (DEGs) were identified using the DESeqR package functions estimate Size Factors and nbinom Test. A *p*‐value < 0.05 and foldchange > 2 or foldchange < 0.5 were set as the threshold for significantly differential expression. Hierarchical cluster analysis of DEGs was performed to explore gene expression patterns. GO enrichment and KEGG pathway enrichment analyses of DEGs were separately performed using R based on the hypergeometric distribution.

## Author Contributions

**Yunwen Qian**, **Yalan Wan**, **Chen Chen** and **Xiaoyu Zheng**: conceptualization, methodology, investigation, data curation, visualization, writing – original draft, writing – review and editing. **Wenjing Du**: methodology, investigation, data curation. **Junhan Yang**: investigation, visualization. **Zhihao Quan**, **Biyu Yang**, **Jiaxi Yu**, **Jie Zheng**, and **Zhaoxia Wang**: investigation, resources. **Jianwen Deng** and **Qiang Guo**: conceptualization, supervision, project administration, funding acquisition, resources, writing – original draft, writing – review and editing.

## Conflicts of Interest

The authors declare no conflicts of interest.

## Supporting information




**Supporting File**: advs76603‐sup‐0001‐SuppMat.docx.

## Data Availability

The representative cryo‐electron tomography datasets supporting the findings of this study have been deposited to EMDB under the following accession codes: EMD‐81544, EMD‐81545, EMD‐81546, EMD‐81547, EMD‐81548, and EMD‐81552. Additional data supporting the findings of this study are available from the corresponding authors upon reasonable request.
