# Supplementary material for: Structural Polymorphism of polyG Inclusions Revealed by In Situ Cryo‐Electron Tomography
Source: Adv Sci (Weinh). 2026 Jul 14;13(56):e76603. doi: 10.1002/advs.76603 (PMC13367101; doi:10.1002/advs.76603)
Supplement: Supplementary file 1 [file ADVS-13-e76603-s001.docx]

**Structural polymorphism of polyG inclusions revealed by *in situ* cryo-electron tomography**

Yunwen Qian^1,#^, Yalan Wan^2,3,#^, Chen Chen^1,4,#^, Xiaoyu Zheng^1,#^, Wenjing Du^1^, Junhan Yang^1^, Zhihao Quan^2,3^, Biyu Yang^5^, Jiaxi Yu^2,3^, Jie Zheng^5,6^, Zhaoxia Wang^2,3^, Jianwen Deng^2,3,^*, Qiang Guo^1,4,^*

1. State Key Laboratory of Membrane Biology, Peking-Tsinghua Joint Center for Life Sciences, Academy for Advanced Interdisciplinary Studies, School of Life Sciences, Peking University, 100871, Beijing, China

2. Department of Neurology, Peking University First Hospital, Beijing 100034, China.

3. Rare Diseases Medical Center, Peking University First Hospital, Beijing, 100034, China.

4. Changping Laboratory, Beijing, China

5. Neuroscience Research Institute and Department of Neurobiology, School of Basic Medical Sciences, Peking University, Beijing, China

6. Key Laboratory for Neuroscience, Ministry of Education/National Health Commission, Peking University, Beijing, 100083, China

#These authors contributed equally to this work.

*To whom correspondence should be addressed. E-mail: [jianwendeng@pkufh.com](mailto:jianwendeng@pkufh.com) (J. D.), [guo.qiang@pku.edu.cn](mailto:guo.qiang@pku.edu.cn) (Q. G.)

**Figure S1. Subcellular distribution of GFP-positive inclusions in primary cortical neurons.**

(a) Representative fluorescence image of primary neurons expressing the polyG–GFP construct. Nuclei are labeled with DAPI (blue) and inclusions are labeled by GFP (green). Scale bar, 50 μm.

(b–d) Enlarged views of the boxed regions in (a) showing representative intranuclear (b), cytoplasmic (c, d) inclusions. Scale bars, 10 μm.

**Figure S2. Correlative cryo-fluorescence and cryo-ET workflow for cellular samples.**

(a) Cryo-fluorescence overview of an EM grid showing GFP-positive targets (green). Scale bar, 200 μm.

(b) Cryo-SEM image of the same grid following cryo-FIB milling, showing a representative lamella. Scale bar, 200 μm.

(c) Correlative overlay of the GFP fluorescence signal with the electron microscopy overview to guide selection of the region of interest. Scale bar, 4 μm.

(d) Low-magnification cryo-TEM image of the milled lamella used for tilt-series acquisition. Scale bar, 4 μm.

(e) Representative tomographic slice from the region indicated in (d). Scale bar, 200 nm.

**Figure S3. Workflow for 26S proteasome identification and state classification.**

**Figure S4. Heterogeneous proteasome association with polyG inclusions by immunofluorescence.**

(a) Representative immunofluorescence images of primary neurons expressing polyG–GFP. Nuclei are labeled with DAPI (blue), inclusions by GFP (green), and proteasomes by anti-PSMC4 staining (red). Scale bar, 50 μm.

(b,c) Examples of cytoplasmic inclusions showing heterogeneous proteasome colocalization, including inclusions with little or no detectable proteasome signal (b) and inclusions with clear colocalization (c).

(d) Representative nuclear inclusions showing proteasome signal enrichment/colocalization.

(e) Control neurons processed in parallel.

Panels (b-e) are shown at the same magnification and share the scale bar shown in (b), 10 μm.

**Figure S5. Colocalization of polyG (4D12) inclusions and p62 in brain tissue from the NIID mouse model by immunofluorescence.**

(a) Representative immunofluorescence images of mouse brain expressing expanded polyG. Nuclei are labeled with DAPI (blue), polyG inclusions by 4D12 (red) and p62 (green). Scale bar, 10 μm.

(b,c) Examples of nuclear inclusions. Scale bar, 10 μm.

(d) Representative cytoplasmic inclusions.

**Figure S6. Workflow for high-pressure freezing, cryo-CLEM, and cryo-FIB lift-out preparation of mouse brain tissue.**

(a) Schematic overview of mouse brain tissue preparation before high-pressure freezing. C57 mouse brains were dissected after perfusion with ice-cold NMDG–HEPES buffer. Coronal brain slices with a thickness of 200 μm were prepared using a vibratome, and tissue blocks from the hippocampal dentate gyrus region were isolated with a biopsy punch.

(b) Schematic illustration of tissue loading onto EM grids for high-pressure freezing. Tissue pieces were placed onto the grid, blotted, assembled into the high-pressure freezing carrier system, and supplemented with 2-methylpentane as the filler before freezing.

(c) Schematic illustration of post-freezing grid recovery. Frozen grids and carriers were transferred into a liquid ethane/propane mixture at −170°C. After removal of 2-methylpentane, the tissue-loaded grids were recovered for subsequent cryo-FIB processing.

(d) Low-magnification cryo-FIB/SEM image acquired at the electron-beam view showing vitrified brain tissue on the grid. The white box indicates the selected tissue block. Scale bar, 200 μm.

(e) Focused ion beam image of the selected tissue block after flattening of the tissue surface. The tissue block corresponds to the boxed region in (d). The inset shows the corresponding EGFP fluorescence signal from the top region of the tissue block. Scale bar, 50 μm.

(f) Cryo-FIB/SEM image showing the selected tissue region for lift-out preparation. Scale bar, 50 μm.

(g) Cryo-FIB/SEM image showing extraction of the tissue block using a nanomanipulator. Scale bar, 10 μm.

(h) Cryo-FIB/SEM image showing transfer of the lifted-out tissue block. Blue arrows indicate the anti-deposition regions along the transfer direction. Scale bar, 20 μm.

(i) Scanning electron microscopy image of the tissue lamella during thinning. The inset shows the corresponding EGFP fluorescence signal from the same region. Scale bar, 5 μm; inset scale bar, 10 μm.

(j) Focused ion beam image of the tissue lamella after thinning. Scale bar, 10 μm.

(k) Overview image showing the outcome of the tissue lift-out procedure on the cryo-FIB grid. Scale bar, 100 μm.

**Figure S7. Cryo-ET of synaptic regions in mouse brain tissue with and without inclusions.**

(a–c) Synaptic region without detectable inclusions. (a) Correlative electron microscopy and fluorescence image of a synaptic area lacking inclusion-associated fluorescence. Scale bar, 5 μm. (b) Representative tomographic slice from the boxed region in (a). Scale bar, 150 nm. (c) Three-dimensional rendering of the tomogram shown in (b). Ribosomes are in yellow, plasma membrane is in grey, synaptic vesicles are in orange, mitochondrion is in bright yellow, myelin sheath is in blue. Scale bar, 150 nm.

(d–f) Synaptic region containing inclusions. (d) Correlative electron microscopy and fluorescence image of a synaptic area with inclusion-associated fluorescence. Scale bar, 5 μm. (e) Representative tomographic slice from the boxed region in (d). Scale bar, 150 nm. (f) Three-dimensional rendering of the tomogram shown in (e). Scale bar, 150 nm.

**Figure S8. Distinct inclusion repertoires across experimental systems.**

(a,b) Representative tomographic views of inclusions in U2OS cells, showing a nuclear inclusion (a) and a cytoplasmic inclusion (b). Scale bars, 150 nm.

(c–e) Representative tomographic views of inclusions in primary neurons, showing the nuclear ribbon network (c), a Cyto1-type cytoplasmic inclusion (d), and a Cyto2-type cytoplasmic inclusion (e). Scale bars, 150 nm.

(f–h) Representative tomographic views of inclusions in mouse brain tissue, showing a nuclear inclusion (f), a Cyto1-like cytoplasmic inclusion (g), and a Cyto2-like cytoplasmic inclusion (h). Scale bars, 150 nm.

**Figure S9 Colocalization of proteasomes with p62-positive inclusions in the brain of NIID mouse model.**

(a) Representative immunofluorescence images of mouse model expressing expanded polyG. Nuclei are labeled with DAPI (blue), inclusions by p62 (red), and proteasomes by PSMC4 (green). Scale bar, 10 μm.

(b-d) Examples of cytoplasmic and nuclear inclusions. Scale bar, 10 μm.

**Figure S10 RNAseq of the brain tissues of NIID mouse model showed significant dysregulation of genes involved in the endoplasmic reticulum (ER) stress response pathways.**

(a) Heatmap showing hierarchical clustering of the differentially expressed mRNAs.

(b) Volcano plots of significantly differentially expressed genes. The criteria of corrected p-value < 0.05 and |Fold of Change| ≥ 2; red, up-regulated; blue, down-regulated.

**Figure S11. Congo red staining of p62-positive inclusions in skin sections from NIID patients.**

Representative skin tissue sections from three NIID patients. The upper row shows immunofluorescence staining for p62 and DAPI, revealing p62-positive intranuclear inclusions. The lower row shows Congo red staining results from NIID patient skin sections. Each column represents one patient. Scale bar, 20 μm.

| Tomogram number | Proteasome concentration in nuclear inclusions (μM) | Proteasome concentration in surrounding nucleoplasm (μM) |
| --- | --- | --- |
| 1 | 0.6834 | 0 |
| 2 | 0.6012 | 0.0371 |
| 3 | 0.3916 | 0.0882 |
| 4 | 0.9506 | 0.052 |
| 5 | 0.6191 | 0.0202 |
| Mean | **0.6492** | **0.0395** |

**Table S1. Concentration (μM) of proteasomes inside and outside nuclear inclusions.**

| Tomogram number | Proteasome concentration in Cyto1 inclusions (μM) | Proteasome concentration in Cyto2 inclusions (μM) |
| --- | --- | --- |
| 1 | 0.2399 | 1.0281 |
| 2 | 0.2459 | 1.0858 |
| 3 | 0.3928 | 1.3879 |
| 4 | 0.3586 | 1.6061 |
| 5 | 0.4878 | 1.3956 |
| 6 | 0.2751 | 1.4207 |
| 7 | 0.2532 | 1.4669 |
| 8 | 0.3826 | 1.5698 |
| Mean | **0.3295** | **1.3701** |

**Table S2. Concentration (μM) of proteasomes within Cyto1 and Cyto2 cytoplasmic inclusions.**
